# Rapid Plasma Synthesis of Gold Nanoparticles Supported on MWCNTs for Electrochemical Detection of Glucose

**DOI:** 10.3390/ma18133076

**Published:** 2025-06-28

**Authors:** Qing Yang, Yuanwen Pang, Hong Li, Lanbo Di

**Affiliations:** 1 College of Physical Science and Technology, Dalian University, Dalian 116622, China; y15128587660@163.com (Q.Y.); pangyuanwen2021@163.com (Y.P.); 2 Key Laboratory of Materials Modification by Laser, Ion and Electron Beams, Dalian University of Technology, Ministry of Education, Dalian 116024, China

**Keywords:** cold plasma, gold nanoparticles, MWCNTs, electrochemical detection of glucose

## Abstract

In this study, a simple, mild, and eco-friendly cold plasma-solution interaction method is employed to rapidly prepare gold colloids. Through modification with multi-walled carbon nanotubes (MWCNTs), a non-enzymatic glucose-sensing electrode material is successfully fabricated. The prepared electrode material is characterized via X-ray diffraction (XRD), Fourier transform infrared spectroscopy (FTIR), X-ray photoelectron spectroscopy (XPS), and transmission electron microscopy (TEM). The results show that compared with the chemically reduced AuNPs-C-MWCNTs, the plasma-prepared AuNPs-P-MWCNTs exhibits enhanced glucose catalytic performance with a higher sensitivity of 73 μA·mM^−1^·cm^−2^ (approximately 3.2 times that of AuNPs-C-MWCNTs), lower response time of 2.1 s, and ultra-low detection limit of 0.21 μM. It also demonstrates excellent selectivity, reproducibility (RSD = 4.37%), repeatability (RSD = 3.67%), and operational stability (RSD = 4.51%). This improvement can be attributed to the smaller particle size and better dispersion of plasma-derived AuNPs on the surface of MWCNTs. Furthermore, the AuNPs-P-MWCNTs surface is enriched with oxygen-containing functional groups, which is conducive to the enhancement of the hydrophilicity of the electrode surface. These synergistic effects facilitate the AuNPs-catalyzed glucose oxidation reaction, ultimately leading to superior glucose catalytic performance.

## 1. Introduction

Glucose, as an essential substance directly involved in metabolic processes, is an important source of energy for the human body. However, chronic overconsumption of glucose can lead to elevated blood glucose levels and cause diabetes and many other health problems [1,2]. The detection and monitoring of glucose levels are not only crucial for the prevention, diagnosis, and treatment of diabetes, but also have far-reaching implications for future preventive medical monitoring, the food industry, the fermentation industry, biopharmaceuticals, and environmental protection [3,4,5]. Therefore, it is crucial to establish a simple, rapid, and accurate method for glucose detection.

With the development of science and technology [6], various types of glucose sensors have been developed based on methods such as fluorescence [7], photoelectrochemical technology [8], enhanced Raman spectroscopy [9,10], and electrochemical analysis [11]. Among them, the electrochemical glucose sensor has outstanding selectivity, sensitivity, and portability and thus has become a mainstream technology for glucose detection [12]. Although traditional enzymatic glucose electrochemical sensors based on biological catalysts have high sensitivity and selectivity, they still have problems such as high cost, poor stability, and poor repeatability due to the inherent defects of glucose oxidase or glucose dehydrogenase [13]. Compared with enzymatic glucose sensors, non-enzymatic glucose sensors are based on direct electrochemical oxidation without the involvement of enzymes, and they have many advantages, such as good stability, good repeatability, and high cost-effectiveness, but at the same time, the high overpotential of glucose oxidation must be overcome. Furthermore, recent advances in non-enzymatic glucose detection using normal body fluids (e.g., saliva: 2.8 μM–1.2 mM, tear: 0.1–0.6 mM, urine: 0–0.8 mM) have demonstrated significant potential for non-invasive medical monitoring applications [14]. However, the glucose concentrations in these biofluids are orders of magnitude lower than blood glucose levels (3–7 mM), posing critical challenges for reliable sensing while simultaneously creating opportunities for early disease screening through ultra-sensitive detection platforms. Therefore, there is an urgent need to develop a catalyst for glucose oxidation with high activity, low detection limits, and low overpotential [15,16].

Noble metal catalysts (e.g., gold, platinum, and palladium) have been widely used for glucose sensing in recent years due to their low redox potential, high selectivity, and freedom from interference by other biomolecules [12]. In particular, gold nanoparticles (AuNPs) are essential materials for the fabrication of non-enzymatic electrochemical sensors due to their enhanced reactivity towards analytes in both neutral and alkaline solutions, better biocompatibility, and lower oxidation potential [17,18].

To prevent the aggregation of gold nanoparticles during the electrocatalytic process, introducing a support material is an essential strategy for obtaining high-performance catalysts [16]. Immobilization of AuNPs on support materials facilitates stabilization of the metallic active sites, enhances their dispersion uniformity, and establishes strong metal–support interactions (SMSIs) for catalytic performance. Carbon-based materials (including carbon quantum dots, carbon nanotubes, carbon fibers, etc.) have high electrical conductivity. They can also be used to immobilize various oxygen-containing functional groups, such as hydroxyl, carboxyl, and epoxy groups, which are excellent support materials for catalytic materials in electrochemical sensors [19]. Among these carbon materials, carbon nanotubes have received significant attention due to their unique properties, such as a distinct tubular structure, high electrical conductivity, excellent chemical and mechanical stability, large specific surface area, etc.

Extensive research has demonstrated that the synergistic interaction between gold nanoparticles and multi-walled carbon nanotubes (MWCNTs) can further enhance the electrocatalytic activity of gold nanoparticles [16,20,21,22,23]. Murugan et al. [23] used mildly oxidized multi-walled carbon nanotubes, gold nanoparticles, and thioglycolic acid to fabricate glucose sensors. The GC-MWCNTs-MSA-AuNPs electrode formed by coating nanohybrids onto a glassy carbon (GC) electrode showed a wide range of responses to glucose concentrations from 0.12–4.0 μM and a low detection limit of 0.036 μM (S/N = 3). Furthermore, the selective sensing of glucose in the presence of H_2_O_2_, uric acid, and a blood cancer drug (imatinib mesylate) was verified through amperometry, and the electrode could be a new addition to glucose sensors and bioanalytical techniques. Branagan et al. [20] dispersed and cast MWCNTs modified with gold nanoparticles onto glassy carbon electrodes and carbon screen-printed electrodes and used them for the detection of glucose in neutral phosphate buffer solution. The electrode exhibits a sensitivity of 2.77 ± 0.14 μA/mM and a limit of detection of 4.1 μM for the linear correction curve, with a linear region extending to 25 mM. The interference of uric acid was successfully eliminated by coating Nafion^®^ film on the composite material, and excellent stability was achieved when the sensor was stored in the air.

Traditional methods for the preparation of noble metal nanoparticles include physical methods [24], chemical reduction [25], and biosynthesis [26], which usually have the disadvantages of expensive equipment and high energy consumption, an environmental pollution risk, and a slow synthesis rate. Compared with these traditional methods, the plasma synthesis process has the advantages of simplicity, rapidity, and environmental friendliness [27,28,29,30]. By exploiting the complex physical and chemical processes, various metal nanoparticles ranging from noble metal NPs to transition metal NPs can easily be synthesized via plasma in a few minutes [31]. Among these plasmas, surface dielectric barrier discharge (SDBD), a typical non-equilibrium plasma operating at atmospheric pressure, enables discharge generation near room temperature without the need for vacuum systems [32,33,34]. During discharge, collisions between high-energy electrons and surrounding gas molecules facilitate excitation, dissociation, and ionization, producing abundant reactive species essential for chemical reactions, such as radicals, excited atoms/molecules, and ions. This technology is particularly effective for synthesizing highly dispersed metal nanoparticles. Its utility stems from the strong reducing capabilities exhibited either by high-energy electrons (typically in inert gases like Ar or He) or by hydrogen species (in hydrogen-containing plasmas, e.g., H_2_/Ar mixtures) [35,36]. Electron reduction predominantly reduces metal ions with positive standard redox potentials (e.g., Au, Ag, Pt, Pd, Ir, Rh). In contrast, hydrogen reduction offers broader applicability and superior efficiency, successfully reducing not only metals with positive redox potentials but also those with negative potentials, such as Co and Ni [37]. For example, Sauvageau et al. [38] used a DBD plasma device to efficiently synthesize Pt, Pd, and Rh nanoparticles from aqueous solutions containing platinum group metal (PGM: Pt, Pd, and Rh) ions, and the technique can also be used to recover PGMs from waste liquids. Du et al. [28] prepared nitrogen/amino co-functionalized MWCNTs-loaded Pd catalysts (Pd/MWCNTs-AP) in solution using a green, mild, and fast SDBD plasma. Structural characterization results showed that Pd/MWCNTs-AP had excellent catalytic performance for formic acid dehydrogenation with ultra-small size Pd, high Pd/C and N/C atom ratios and abundant -OH.

When plasma interacts with liquids, it can trigger complex physical and chemical reactions at the gas–liquid interface, generating a large number of chemically active particles [31,39,40]. Typically, these active particles include short-lived particles such as free electrons, hydrated electrons (e^−^_aq_), excited hydrogen atoms (H), negative hydrogen ions (H^−^), and long-lived particles such as H_2_O_2_. In addition, alcohol fragmentation radicals induced by high-energy electrons or UV radiation are generated when ethanol is added to the solution. These reactive radicals have a lower redox potential and can effectively reduce the noble metal ions to elemental nanoparticles [41]. Bjelajac et al. [42] proposed a one-step synthesis method for well-dispersed AuNPs. They atomized pure ethanol, which served as the solvent for the gold precursor, in an atmospheric pressure dielectric barrier discharge (DBD) plasma torch, forming a carbon-based matrix around the AuNPs and obtaining well-dispersed AuNPs. Mariotti et al. [43] discussed the successful synthesis of AuNPs by directly or indirectly reducing the HAuCl_4_ aqueous precursor solution with electrons generated from the interaction between plasma and liquid without additional reducing agents, surfactants, or capping agents. Yang et al. [44] directly synthesized Au/CuO nanoparticles with particle sizes in the range of 20–40 nm using the solution plasma method and successfully applied them to the non-enzymatic detection of glucose.

This study demonstrates a simple, mild, and green plasma–liquid interaction strategy to prepare gold colloids and then construct carbon material-supported gold nanoparticles for non-enzymatic glucose sensing, focusing on the effects of the addition sequence, type, and amount of carbon materials, the concentration of HAuCl_4_ precursor, as well as the discharge time and discharge voltage on the glucose-sensing performance. The results show that the atmospheric pressure surface dielectric barrier discharge (SDBD) plasma system, operated at 6 kV with H_2_/Ar (volume ratio of 1:1) as the working gas, enables rapid synthesis of gold colloids through 7 min treatment of 0.5 mM HAuCl_4_ in ethanol aqueous solution (50% water, 50% ethanol). The AuNPs-P-MWCNTs nanocomposite, synthesized via subsequent addition of 10 mg MWCNTs, exhibits outstanding performance with a sensitivity of 73 μA·mM^−1^·cm^−2^, response time of 2.1 s, and detection limit of 0.21 μM. Compared with the AuNPs-C-MWCNTs prepared using the traditional chemical reduction method, this plasma method produces smaller-sized AuNPs with uniform dispersion on the surface of MWCNTs, which is conducive to the catalytic oxidation of glucose by the AuNPs, and significantly improves the catalytic activity.

## 2. Experimental Section

### 2.1. Materials

Gold trichloride (AuCl_3_·HCl·4H_2_O, AR), glucose (C_6_H_12_O_6_·H_2_O, AR), potassium hydroxide (KOH, AR, ≥85.0%), sodium citrate (C_6_H_5_Na_3_O_7_·2H_2_O, HPLC, ≥99.5%), and anhydrous ethanol (AR, ≥99%) used in this experiment were purchased from Kermel Chemical Reagent Co., Ltd. (Tianjin, China). MWCNTs and graphite oxide (GO) powder were purchased from Xianfeng Nanomaterials Technology Co., Ltd. (Nanjing, China). (The MWCNTs feature length: 0.5–2 μm, ID: 2–5 nm, OD: 5–15 nm, wall count: ~15, purity: >95%). Apricot shell carbon (AC) was purchased from Guanghua Wood Factory (Beijing, China). Superconducting carbon black (CB) was purchased from Aiweixin Chemical Technology Co., Ltd. (Tianjin, China). Graphene (GR) was purchased from the Carbon Materials Test Network. High-purity argon (>99.999%) and hydrogen (>99.999%) were provided by Zhonghao Guangming Chemical Research and Design Institute Co., Ltd. (Dalian, China).

### 2.2. Catalyst Preparation

#### 2.2.1. Preparation of AuNPs-P-MWCNTs

Firstly, gold colloids were successfully prepared by treating 0.5 mM HAuCl_4_ ethanol aqueous solution containing 50% ethanol with SDBD cold plasma using a mixture of H_2_ and Ar (volume ratio of 1:1) as the working gas at a total flow rate of 100 SCCM. The SDBD reactor consists of a high-voltage electrode and a grounding electrode, which are separated by a high-purity alumina dielectric layer (area 9 × 5 cm^2^, thickness 1 mm). Both electrodes are made of high-purity tungsten. The high-voltage electrode consists of nine comb tungsten wires (tungsten filament width of 1 mm, filament spacing of 4 mm) connected at one end, and the size of the grounding electrode is 1.7 × 0.5 cm^2^. The voltage across them was measured by a high-voltage probe (Tektronix P6015A, Beaverton, OR, USA). The rotational speed of the magnetic stirrer was operated at 500 rpm, while applying a peak-to-peak voltage of 6.0 kV at the discharge frequency of 10.4 kHz, and the plasma treatment was sustained for 7 min. After the plasma treatment, a wine-red solution was obtained. The solution was poured into a 5 mL measuring cylinder, and ethanol was added to compensate for the liquid lost during plasma treatment to a total volume of 2 mL. After mixing well, it was poured into the sample bottle and recorded as AuNPs-P. Then, 1 mL of freshly prepared gold colloid was mixed with 10 mg of multi-walled carbon nanotubes, which was denoted as AuNPs-P-MWCNTs. Subsequently, 40 μL of Nafion solution was added dropwise and mixed, ultrasonically dispersed, and applied dropwise to the electrode for use.

#### 2.2.2. Preparation of AuNPs-C-MWCNTs

The AuNPs-C-MWCNTs nanocomposite was synthesized via a hydrothermal method. First, 1.25 mL of HAuCl_4_ solution (20 mM) was placed in a round-bottomed flask, adjusted to 95 mL with deionized water, heated to boiling, and 5 mL of 1% sodium citrate solution was added to the boiling solution under mechanical agitation. After adding sodium citrate, the solution rapidly turned blue-purple and changed to wine-red within a few seconds. After continuous stirring for 5 min, a final 0.25 mM of gold colloids were obtained [45,46]. Then, 1 mL of freshly prepared gold colloid was mixed with 5 mg of multi-walled carbon nanotubes (keeping the ratio of Au and multi-walled carbon nanotubes consistent with that of the AuNPs-P-MWCNTs catalytic material), which was denoted as AuNPs-C-MWCNTs. Subsequently, 40 μL of Nafion solution was added and mixed, followed by ultrasonic dispersion and drop-coating onto the electrode for use.

#### 2.2.3. Preparation of Electrodes

Aliquots of 10 μL (AuNPs-P-MWCNTs) and 20 μL (AuNPs-C-MWCNTs) colloidal suspensions were precisely pipetted after ultrasonic treatment, respectively (keeping the Au content consistent) and deposited onto the surface of the polished glassy carbon electrodes. After drying at 25 °C, glassy carbon electrodes modified with gold nanoparticles and carbon materials were obtained. By altering the addition sequence, type, and amount of carbon materials, the concentration of HAuCl_4_ precursor, and the plasma discharge parameters, different types of modified electrodes can be obtained.

### 2.3. Catalyst Characterization and Electrochemical Testing

The functional groups on the catalyst surface were characterized using a Fourier transform infrared spectrometer (FTIR) (Nicolet iS20, Thermo Scientific, Waltham, MA, USA). An X-ray diffractometer (XRD) (DX-2700, Dandong, China) was used to generate Cu-Kα radiations (λ = 1.54178 Å) at a tube voltage of 40 kV and tube current of 30 mA to characterize the species composition and the crystal phase structure of the samples. X-ray photoelectron spectroscopy (XPS, Thermo Scientific Model K-Alpha, Waltham, MA, USA) was used to study the surface composition and chemical state of the materials, and all spectra were calibrated with a C 1s spectrum at 284.8 eV. The microstructure of the samples was observed via a transmission electron microscope (TEM) (JEOL JEM-2100F, Tokyo, Japan) at an accelerating voltage of 120 kV. By analyzing at least 100 nanoparticles in the TEM images, the average particle size of the AuNPs can be determined. The glucose-sensing performance of the modified glassy carbon electrode was tested on an electrochemical workstation (CHI 760E, Shanghai, China), and different test methods were employed, including cyclic voltammetry (CV) (voltage range of −0.8 to 0.8 V vs. Hg/HgO, scan rate of 100 mV·s^−1^), amperometric (i-t) (glucose assay with applied voltage of 0.1 V vs. Hg/HgO), and electrochemical impedance spectroscopy (EIS) (electrolyte of 0.1 M KOH, applied amplitude of ±5 mV, frequency range of 0.1 to 10^5^ Hz). The double-layer capacitance (C_dl_) was determined by recording CV curves within a non-Faradic potential window (−0.8 V to −0.7 V vs. Hg/HgO) in 0.1 M KOH electrolyte at scan rates ranging from 20 to 120 mV·s^−1^. For each scan rate, the current differential Δ*j* = |*j*_a_ − *j*_c_| at −0.75 V was calculated and the C_dl_ value was estimated by fitting the slope of the Δ*j* vs. the scan rate plot. A standard three-electrode system was used for the experiments, with the modified glassy carbon electrode acting as the working electrode, the Hg/HgO and platinum wire electrodes as the reference and counter electrodes, respectively, and the electrolyte was 0.1 M KOH solution. The diameter of the glassy carbon electrode used is 3 mm.

## 3. Results and Discussion

### 3.1. Electrochemical Properties

First, the addition of MWCNTs is studied via cyclic voltammetry and amperometric methods, as shown in Figures S1 and S2, in which AuNPs-P denotes treatment of HAuCl_4_ precursor using argon-hydrogen cold plasma directly, AuNPs-P-MWCNTs represents plasma treatment of HAuCl_4_ precursor to obtain AuNPs-P followed by addition of MWCNTs without discharge, and AuNPs/MWCNTs-P indicates plasma treatment of a mixture of MWCNTs and HAuCl_4_ solution. Then, the effects of the type of carbon material (MWCNTs, GO, GR, CB, and AC in Figures S3 and S4), amount of MWCNTs (5 mg, 10 mg and 15 mg in Figures S5 and S6), concentration of HAuCl_4_ (0.25 mM, 0.5 mM and 1 mM in Figures S7 and S8), discharge time (3 min, 7 min and 9 min in Figures S9 and S10), and discharge voltage (4 kV, 6 kV and 9 kV in Figures S11 and S12) on the performance of gold-based catalytic materials toward glucose detection are systematically investigated. The results show that AuNPs-P-MWCNTs prepared via plasma treatment of 0.5 mM HAuCl_4_ ethanol aqueous solution containing 50% ethanol at 6 kV for 7 min with a mixed gas of hydrogen and argon as working gas, followed by the addition of 10 mg of MWCNTs, exhibits the optimal glucose-sensing performance (Figures S1–S12).

**Figure 1:**
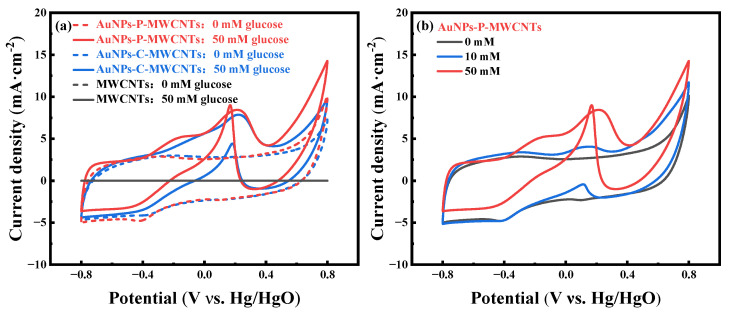
(**a**) CV curves of AuNPs-P-MWCNTs, AuNPs-C-MWCNTs, and MWCNTs in 0.1 M KOH with 0 mM and 50 mM glucose at a scan rate of 100 mV·s^−1^ and (**b**) CV curves of AuNPs-P-MWCNTs in 0.1 M KOH with 0, 10, and 50 mM glucose at a scan rate of 100 mV·s^−1^.

Figure 1a shows the cyclic voltammetry (CV) curves of AuNPs-P-MWCNTs prepared via the plasma method, AuNPs-C-MWCNTs prepared via the chemical reduction method, and MWCNTs, in the absence (dotted line) and presence (solid line) of 50 mM glucose at a scan rate of 100 mV·s^−1^, respectively. As can be seen from the figure, there is no redox peak on pristine MWCNTs with and without glucose, indicating the negligible catalytic performance of MWCNTs within the applied potential range [47]. In contrast, the CV curves of AuNPs-P-MWCNTs and AuNPs-C-MWCNTs exhibit weak redox peaks in the absence of glucose, and their CV curves almost overlap. When 50 mM glucose is added to the electrolyte, distinct redox peaks appear in the CV curves of both electrodes. Although the oxidation potentials of both electrodes are close to each other in the CV curves, the AuNPs-P-MWCNTs exhibits higher anodic oxidation peak current density compared to AuNPs-C-MWCNTs. Moreover, the cathodic peak current density of AuNPs-P-MWCNTs shows more prominent enhancement during the reverse scan, providing superior electrocatalytic activity for glucose.

To further investigate the electrocatalytic behavior of the AuNPs-P-MWCNTs-modified electrode toward glucose oxidation, its CV curves at different glucose concentrations are tested, as shown in Figure 1b. It can be seen that the oxidation peak current increases and the peak potential shifts gradually with the increase of glucose concentrations, indicating the presence of a large number of electroactive species at the electrode surface with good electrocatalytic activity for electrochemical non-enzymatic glucose oxidation [48,49]. The mechanism of direct glucose oxidation is closely related to the formation of gold hydroxide Au [(OH)_ads_) sites [22,50], which are formed as a result of OH^−^ adsorption onto the surface of gold nanoparticles. When introducing 10 mM glucose in the electrolyte, as shown in Figure 1b, an oxidation peak is first observed at about −0.35 V during a positive scan. The emergence of this minor peak signifies OH^−^ adsorption onto the gold surface, forming the Au (OH)_ads_ active sites (Equation (1)). Simultaneously, glucose is also adsorbed onto the gold surface to generate the intermediate gluconolactone (Equation (2)). With the progressive formation of Au (OH)_ads_, sustained catalytic oxidation of glucose occurs, generating an intensified anodic oxidation peak at about 0.15 V. As the reaction proceeds, the generated gold oxides occupy the active sites, leading to a decrease of Au (OH)_ads_ density and thereby inhibiting the glucose oxidation process. During the reverse scan, a strong cathodic oxidation peak emerges around 0.1 V, which can be ascribed to the reduction of gold oxides, regenerating new Au (OH)_ads_ active sites (Equation (3)). Thus, this redox cycling replenishes active sites for glucose re-adsorption and subsequent oxidation [15,18]. The electrocatalytic mechanism of glucose on AuNPs-P-MWCNTs presumably involves the following continuous redox reactions [16,51]:

(1) $$Au+{OH}^{-}\to {Au\left(OH\right)}_{ads}+e^{-}$$

(2) $${Au\left(OH\right)}_{ads}+glucose\to Au+H_{2}O+gluconolactone$$

(3) $${Au(OH)}_{ads}⇌{Au}_{2}O_{3}+H_{2}O$$

**Figure 2:**
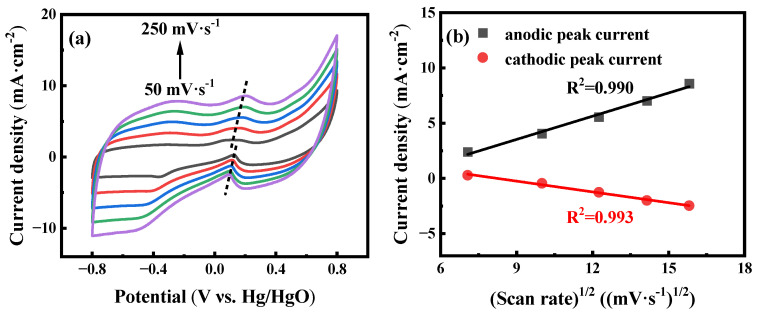
(**a**) CV curves of AuNPs-P-MWCNTs in 0.1 M KOH with 10 mM glucose at different scan rates ranging from 50 to 250 mV·s^−1^ (from **bottom** to **up**) with a step rate of 50 mV·s^−1^ and (**b**) linear fitting of the corresponding peak current to the square root of the scan rate.

The influence of scan rates is also evaluated to investigate the electrochemical reaction kinetics of the AuNPs-P-MWCNTs-modified electrode. Figure 2a shows the CV responses of the AuNPs-P-MWCNTs electrode with scan rates ranging from 50 to 250 mV·s^−1^ with addition of 10 mM glucose in 0.1 M KOH solution. It can be seen that the current density of the glucose oxidation peak increases monotonically with increasing scan rates, accompanied by a positive shift in the anodic peak and a negative shift in the cathodic peak, confirming a rapid and reversible process of glucose oxidation [52]. Furthermore, the current density of the oxidation peak depicts a linear relationship with the square root of the scan rate (R^2^ = 0.990 and 0.993), following a Randles–Sevcik equation, as shown in Figure 2b. This linear relationship indicates that the electron transfer of the AuNPs-P-MWCNTs-modified electrode is a typical diffusion-controlled electrochemical process. [12,22].

**Figure 3:**
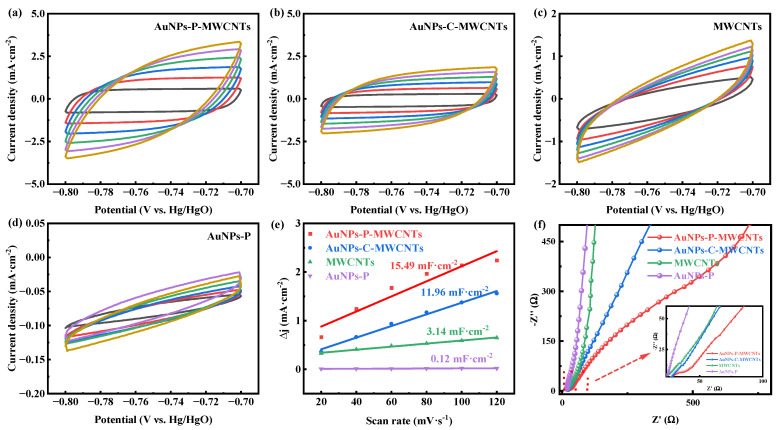
CV curves of (**a**) AuNPs-P-MWCNTs, (**b**) AuNPs-C-MWCNTs, (**c**) MWCNTs, and (**d**) AuNPs-P with the scan rate ranging from 20 to 120 mV·s^−1^ in 0.1 M KOH, and (**e**) the calculated double-layer capacitance (C_dl_) for different materials; (**f**) Nyquist plots of AuNPs-P-MWCNTs, AuNPs-C-MWCNTs, MWCNTs, and AuNPs-P in 0.1 M KOH.

In order to explain the electrochemical behavior of the different modified electrodes, their electrochemical active surface areas (ECSAs) and electrochemical impedance are investigated. Figure 3a–d show the CV curves of AuNPs-P-MWCNTs, AuNPs-C-MWCNTs, MWCNTs, and AuNPs-P, respectively, in 0.1 M KOH solution at different scan rates. According to these CV curves, the double layer capacitance (C_dl_) of different modified electrodes can be calculated, where a larger C_dl_ indicates a larger ECSA [53]. As shown in Figure 3e, the measured C_dl_ values are 15.49, 11.96, 3.14, and 0.12 mF·cm^−2^, respectively, following the order of AuNPs-P-MWCNTs > AuNPs-C-MWCNTs > MWCNTs > AuNPs-P. This result indicates that the AuNPs-P-MWCNTs electrode possesses the highest ECSA, which can provide abundant active sites for glucose oxidation reactions. Furthermore, Figure 3f presents the Nyquist plots of these modified electrodes. The AuNPs-P-MWCNTs electrode exhibits the smallest semicircle diameter in the high-frequency region, demonstrating its smallest charge transfer resistance. This lower resistance facilitates electron transfer and consequently accelerates the electrochemical reaction kinetics.

**Figure 4:**
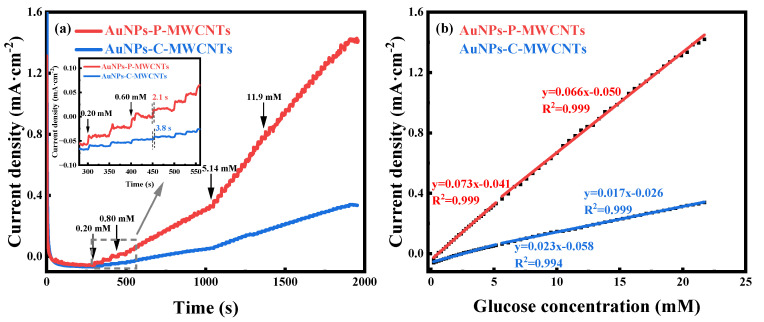
(**a**) Amperometric responses of AuNPs-P-MWCNTs and AuNPs-C-MWCNTs with continuous addition of glucose at the optimal catalytic potential of 0.1 V vs. Hg/HgO and (**b**) the corresponding linear fitting of current density versus glucose concentration for both electrodes. Inset (**a**) presents the response time of both electrodes with addition of 0.8 mM glucose.

The amperometric responses of both the AuNPs-P-MWCNTs and the AuNPs-C-MWCNTs electrodes with continuous addition of glucose at the optimal working potential of 0.1 V are depicted in Figure 4a; the inset shows the response times of both electrodes with addition of 0.8 mM glucose. The optimal working potentials of the two electrodes are obtained from Figures S12b and S13. By comparison, the AuNPs-P-MWCNTs electrode exhibits a remarkably higher step-current response and lower response time (as low as 2.1 s). Figure 4b illustrates the linear fitting of the current density with the glucose concentration for both the AuNPs-P-MWCNTs and the AuNPs-C-MWCNTs electrodes, where the slope represents the sensitivity of the electrode materials. The sensitivity of the AuNPs-P-MWCNTs electrode is 73 μA·mM^−1^·cm^−2^ (0.2–5.15 mM) at low glucose concentration and 66 μA·mM^−1^·cm^−2^ (5.64–21.72 mM) at high glucose concentration, both with good linearity (R^2^ = 0.999 and 0.999). Based on the obtained sensitivity, the detection limit is calculated to be 0.21 μM using the equation,

(4) $$LOD=\frac{3S}{N}$$

where S represents the standard deviation of the electrode’s current in 0.1 M KOH at 0.1 V vs. Hg/HgO, and N is the sensitivity of the electrode. Similarly, the sensitivity of the AuNPs-C-MWCNTs electrode can be calculated to be 23 μA·mM^−1^·cm^−2^ and 17 μA·mM^−1^·cm^−2^ for both low and high glucose concentrations, respectively, with similarly good linearity (R^2^ = 0.999 and 0.998), and a detection limit of 0.17 mM. Therefore, the AuNPs-P-MWCNTs electrode shows higher sensitivity, a lower response time, and a lower detection limit compared to the AuNPs-C-MWCNTs electrode, which reveals outstanding performance of electrocatalytic glucose oxidation.

**Figure 5:**
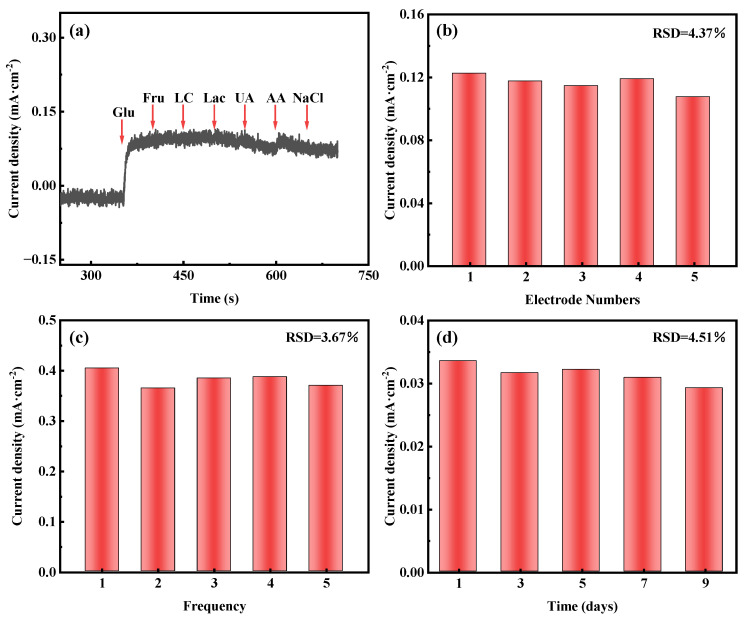
Amperometric responses of AuNPs-P-MWCNTs electrode: (**a**) in 0.1 M KOH with continuous dropwise addition of 2 mM glucose, 0.2 mM fructose, lactose, lactic acid, uric acid, ascorbic acid, and sodium chloride at 0.1 V vs. Hg/HgO, (**b**) reproducibility test using five electrodes prepared identically towards 2 mM glucose, (**c**) repeatability test of one electrode towards 6 mM glucose over five measurements, (**d**) stability test towards 1 mM glucose over 9 days.

In addition to sensitivity and the detection limit, selectivity is also an important parameter for evaluating the performance of non-enzymatic glucose sensors. Glucose is known to be present in human serum along with other interfering chemicals, such as fructose, lactose, lactic acid, uric acid, ascorbic acid, and sodium chloride. At normal physiological values, the concentration of glucose is about 10 times higher than that of other interfering chemicals [54]. We evaluate selectivity by using sequential addition of 2 mM of glucose and 0.2 mM of other interfering chemicals (e.g., fructose, lactose, lactic acid, uric acid, ascorbic acid, and sodium chloride) to 0.1 M KOH at 0.1 V (vs. Hg/HgO). As shown in Figure 5a, a significant current response is observed when 2 mM of glucose is added, in contrast to the addition of other interfering substances with a negligible signal. This result confirms the excellent selectivity of AuNPs-P-MWCNTs for glucose detection.

In order to investigate the reproducibility, repeatability, and stability of the AuNPs-P-MWCNTs electrode, the reproducibility testing is conducted using five identically prepared AuNPs-P-MWCNTs electrodes exposed to 2 mM of glucose, as shown in Figure 5b, yielding a relative standard deviation (RSD) of 4.37% for the response current. The repeatability is assessed by repeatedly testing a single AuNPs-P-MWCNTs electrode towards 6 mM of glucose five times, as shown in Figure 5c, which shows an RSD of 3.67% in the response current. For long-term stability, the response current of one AuNPs-P-MWCNTs electrode to 1 mM of glucose, tested periodically over 9 days, exhibits an RSD of 4.51%, as shown in Figure 5d. These results confirm that the AuNPs-P-MWCNTs electrode exhibits reliable reproducibility, good repeatability, and good stability.

### 3.2. Structure and Morphology of Nanocomposites

**Figure 6:**
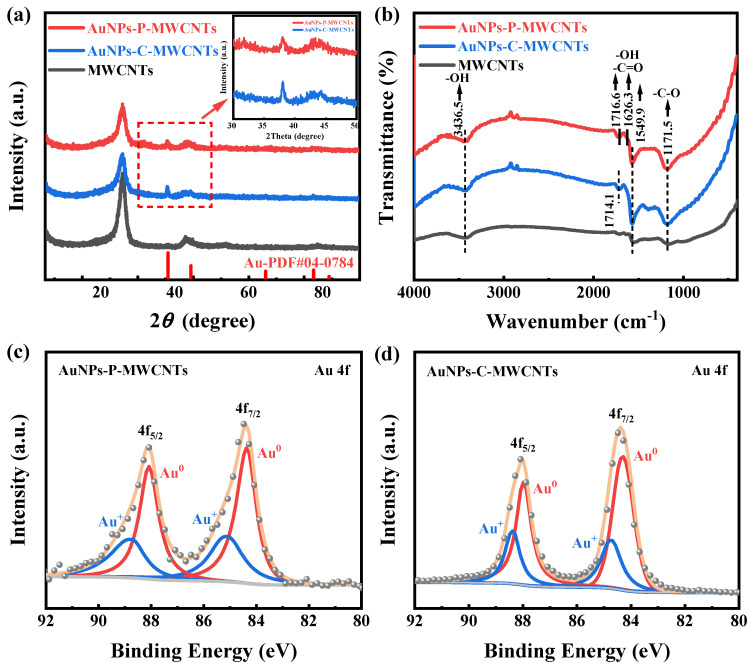
(**a**) XRD patterns and (**b**) FTIR spectra of AuNPs-P-MWCNTs, AuNPs-C-MWCNTs, and MWCNTs; XPS spectra of Au 4f in (**c**) AuNPs-P-MWCNTs, and (**d**) AuNPs-C-MWCNTs.

XRD characterization is performed for AuNPs-P-MWCNTs, AuNPs-C-MWCNTs, and MWCNTs to examine the species composition and phase structures, as shown in Figure 6a. Distinct characteristic diffraction peaks at 38.2° are observed for both AuNPs-P-MWCNTs and AuNPs-C-MWCNTs compared to pristine MWCNTs, corresponding to the (111) plane of the face-centered cubic crystal structure of metallic Au (PDF#04-0784), which confirms the generation of AuNPs for both the plasma-synthesized and chemically reduced catalysts. In addition, the AuNPs-C-MWCNTs exhibits a slightly higher diffraction peak at 38.2°, which is attributed to the greater reduction of Au ions using the chemical reduction method justified by the XPS result. Figure 6b shows the FTIR spectra of AuNPs-P-MWCNTs, AuNPs-C-MWCNTs, and MWCNTs. The broad absorption at 3436.5 cm^−1^ corresponds to the stretching vibration of -OH, the spectral envelope spanning from 1545 cm^−1^ to 1720 cm^−1^ includes overlapping bands from -C=O stretching (1680–1720 cm^−1^) and -OH bending modes (1545–1640 cm^−1^), and the distinct peak at 1171.5 cm^−1^ arises from the stretching vibration of -C-O. It can be seen from the figure that the surface of the original MWCNTs contains many oxygen-containing functional groups. Simultaneously, compared with MWCNTs, the peaks of the oxygen-containing functional groups (-OH, -C=O, -C-O) of AuNPs-C-MWCNTs and AuNPs-P-MWCNTs exhibit increased intensities. The enriched functional groups are conducive to the enhancement of hydrophilicity of the catalytic material, which facilitates the contact between the electrode surface and reactants, thus improving the catalytic activity [28,55,56].

XPS characterization is also conducted to further study the chemical valence of AuNPs-P-MWCNTs and AuNPs-C-MWCNTs, as shown in Figure 6c,d. The fitted peaks located at 84.32 eV and 88.02 eV correspond to the 4f_7/2_ and 4f_5/2_ regions of Au^0^, respectively [22,57]. The presence of Au^0^ indicates that both plasma and chemical reduction can reduce gold ions to metallic gold monomers. However, due to the existence of oxygen-containing functional groups on the surface of MWCNTs, the gold precursors in AuNPs-P-MWCNTs and AuNPs-C-MWCNTs are not completely reduced and exist in the metallic state Au^0^ and oxidized state Au^+^ [27,28,35]. Based on a semi-quantitative analysis of the samples, the proportion of Au^0^ in AuNPs-P-MWCNTs and AuNPs-C-MWCNTs is approximately 64% and 69%, respectively, indicating a higher degree of reduction of gold ions using the chemical reduction method. Indeed, plasma is a mild treatment method with lower reduction of gold ions and better dispersion of the prepared gold nanoparticles on carbon nanotubes [28], which is consistent with the XRD analysis results.

**Figure 7:**
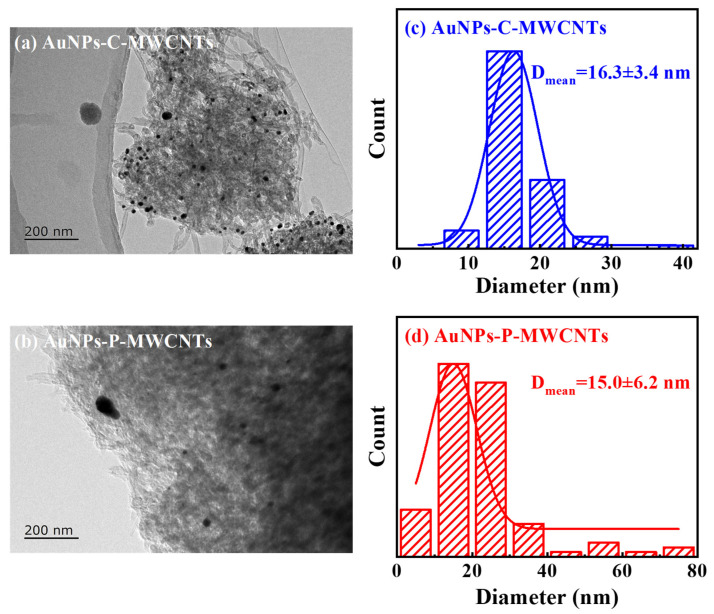
TEM images of (**a**) AuNPs-C-MWCNTs and (**b**) AuNPs-P-MWCNTs and (**c**,**d**) histograms of the particle size distribution of the corresponding Au nanoparticles.

The TEM images and histograms of the particle size distribution of AuNPs-C-MWCNTs and AuNPs-P-MWCNTs are given in Figure 7, respectively. As can be seen in Figure 7a, Au nanoparticles are clearly visible on the outer surface of the MWCNTs, indicating surface-bound AuNPs [22] in AuNPs-C-MWCNTs with a narrow size distribution (the average particle size of Au nanoparticles is 16.3 ± 3.4 nm), demonstrating high colloidal stability from chemical reduction. In contrast, gold nanoparticles in AuNPs-P-MWCNTs exhibit various sizes and are uniformly distributed on the surface of multi-walled carbon nanotubes (the average particle size of gold nanoparticles is about 15.0 ± 6.2 nm), which is slightly larger than that of the gold nanoparticles (12.32 ± 0.87 nm) prepared by Kim et al. [58] using a direct current discharge plasma in the presence of PVP and KCl, while it is smaller than Au/CuO nanoparticles (20–40 nm) synthesized by Yang et al. using the solution plasma method [44]. This morphological divergence could originate from different synthesis methods. AuNPs prepared via chemical reduction exhibit great stability, and their particle size does not change after mixing with MWCNTs. In contrast, a small number of AuNPs prepared via the plasma reduction method undergo aggregation in the process of mixing with MWCNTs, due to the lack of protection from surfactants, forming larger-sized AuNPs. Nevertheless, a large number of AuNPs prepared via plasma reduction are able to be well adsorbed and dispersed on the surface of MWCNTs, which effectively inhibits the agglomeration of AuNPs. Since smaller crystal grain sizes typically have a larger specific surface area [59], the AuNPs-P-MWCNTs obtained through plasma reduction is more conducive to the adsorption of OH^−^ onto the surface of gold nanoparticles, providing more active sites for the direct catalytic oxidation of glucose, showing strong glucose catalytic performance [60].

### 3.3. Discussion

This study employs a rapid, mild, and green plasma–solution interaction approach to synthesize gold colloids modified by multi-walled carbon nanotubes (AuNPs-P-MWCNTs), which are compared with chemically reduced counterparts (AuNPs-C-MWCNTs) for electrochemical detection of glucose. XRD analysis confirms the generation of AuNPs for both the plasma-synthesized and chemically reduced method. The FTIR result indicates a significant increase of oxygen-containing functional groups in both composites, where the enriched functional groups are conducive to the enhancement of the hydrophilicity of the electrode surface. XPS analyses show that the plasma is a mild treatment with a lower degree of reduction of the gold ions. TEM analysis with size distribution histograms confirms that H_2_/Ar cold plasma enables the synthesis of smaller AuNPs (15.0 ± 6.2 nm vs. 16.3 ± 3.4 nm) well dispersed on the surface of multi-walled carbon nanotubes, which is more favorable for OH^−^ adsorption onto the surface of AuNPs, providing more active sites for the direct catalytic oxidation of glucose. In summary, AuNPs with smaller particle sizes and uniform dispersion on the surface of MWCNTs enriched with oxygen-containing functional groups can be synthesized using a plasma process, and these synergistic advantages enhance the performance of electrocatalytic glucose oxidation.

A comparison of the glucose detection performance among various gold-based non-enzymatic glucose-sensing electrode materials reported in the literatures is summarized in Table 1. Although the sensitivity of AuNPs-P-MWCNTs (73 μA·mM^−1^·cm^−2^) does not reach the state-of-the-art gold-based catalysts, its ultra-low detection limit of 0.21 μM demonstrates 1–2 orders of magnitude superiority over analogous materials. This exceptional feature is essential for the development of high-precision portable glucose monitoring devices and demonstrates great potential for non-invasive medical monitoring applications.

## 4. Conclusions

This study demonstrates a simple, rapid, and green cold plasma–solution interaction strategy to prepare gold colloids, followed by homogeneous mixture with MWCNTs for the preparation of an electrode (AuNPs-P-MWCNTs), and is compared with the electrode of MWCNTs-supported AuNPs prepared using the conventional chemical reduction method (AuNPs-C-MWCNTs). Experimental results show that AuNPs-P-MWCNTs prepared via SDBD plasma treatment of 0.5 mM HAuCl_4_ ethanol aqueous solution containing 50% ethanol at 6 kV for 7 min with a mixed gas of hydrogen and argon as the working gas, followed by the addition of 10 mg of MWCNTs, exhibits the optimal glucose-sensing performance. The sensitivity of its modified electrode is 73 μA·mM^−1^·cm^−2^ with a response time of 2.1 s and a detection limit as low as 0.21 μM. It also demonstrates excellent selectivity, reproducibility (RSD = 4.37%), repeatability (RSD = 3.67%), and operational stability (RSD = 4.51%). These results indicate that AuNPs-P-MWCNTs exhibits exceptional glucose sensing capabilities, positioning it as a promising electrode material for enzyme-free glucose sensors.

## Figures and Tables

**Table 1:** A comparison of glucose detection performance among various Au-based non-enzymatic glucose sensors reported in the literature.

Electrode Materials	Detection Limit (μM)	Sensitivity (μA·mM^−1^·cm^−2^)	Dynamic Range (mM)	Response Time	Supporting Electrolyte	References
AuNPs/GC	50	87.5	0.1–25.0	/	0.1 M NaOH	[61]
Au NCs/GCE	100	2131	1.0–9.0	/	0.1 M NaOH	[62]
Porous Au	5	11.8	2.0–10.0	2 s	0.1 M PBS + 0.1 M KCl	[63]
Au NPs/ITO	5	23.0	up to 11	/	0.5M KOH	[64]
AuNPs/PANI/CC	3.08	150	0.0126–10.0	/	0.5M KOH	[65]
Pt-Au/MWCNT	10	10.71	0.04–24.4	/	0.01 M PBS	[66]
Au-Gt	66	19.4	1–14	/	0.3 KOH	[67]
Au-rGtO	63	39.8	0–10	/		
Au-GtO	99	98.7	0–25	/		
AuNPs/Ni(OH)_2_ NS	0.66	82.71	0.002–6	/	0.1 M NaOH	[68]
Au-DS/GSE	190	1904	0.05–5	/	0.1 M KOH	[69]
Au-Gr/GE	12	604	0.05–42	/	0.1 M PBS	[70]
Au-graphene nanosheet	50	326	0.1–16	/	0.1 M PBS	[71]
GCE-MWCNTs-MSA-AuNPs	0.036	22.9 μA·cm^−2^	0.00012–0.004	/	KOH	[23]
fn.MWCNT–Au_nano_ modified SPE	4.1	2.77 ± 0.14 μA·mM^−1^	0.1–25.0	/	0.1 M PBS	[20]
Nafion^®^ modified fn.MWCNT–Au_nano_ SPE	10.0	0.55 ± 0.03 μA·mM^−1^	0.1–25.0	/	0.1 M PBS	[20]
AuNPs-C-MWCNTs	170	23	0.2–21.71	3.8 s	0.1 M KOH	This work
AuNPs-P-MWCNTs	0.21	73	0.2–21.71	2.1 s	0.1 M KOH	This work

## Data Availability

The original contributions presented in this study are included in the article/Supplementary Materials. Further inquiries can be directed to the corresponding author.

## Supplementary Materials

The following supporting information can be downloaded at: https://www.mdpi.com/article/10.3390/ma18133076/s1, Figure S1: Cyclic voltammetry (CV) curves of AuNPs-P, AuNPs-P-MWCNTs, AuNPs/MWCNTs-P, and MWCNTs with 0 mM and 50 mM glucose in 0.1 M KOH at a scan rate of 100 mV·s^−1^; Figure S2: Chronoamperometric responses of (a) AuNPs-P, (b) AuNPs-P-MWCNTs, and (c) AuNPs/MWCNTs-P with continuous addition of glucose in 0.1 M KOH at working potentials of −0.15, −0.1, 0, 0.1, 0.15, and 0.2 V vs. Hg/HgO; Figure S3: CV curves of (a) AuNPs-P-MWCNTs, (b) AuNPs-P-GO, (c) AuNPs-P-GR, (d) AuNPs-P-CB, and (e) AuNPs-P-AC with 0 mM and 50 mM glucose in 0.1 M KOH at a scan rate of 100 mV·s^−1^; Figure S4: Chronoamperometric responses of (a) AuNPs-P-MWCNTs, (b) AuNPs-P-GO, (c) AuNPs-P-GR, (d) AuNPs-P-CB, and (e) AuNPs-P-AC with continuous addition of glucose in 0.1 M KOH at working potentials of −0.1, 0, 0.1, 0.15, and 0.2 V vs. Hg/HgO; Figure S5: CV curves of AuNPs-P-MWCNTs with different amounts of MWCNTs (a) 5 mg, (b) 10 mg, and (c) 15 mg with 0 mM and 50 mM glucose in 0.1 M KOH at a scan rate of 100 mV·s^−1^, and (d) comparisons of CV curves of AuNPs-P-MWCNTs with different amounts of MWCNTs in 0.1 M KOH containing 50 mM glucose at a scan rate of 100 mV·s^−1^; Figure S6: Chronoamperometric responses of AuNPs-P-MWCNTs with different amounts of MWCNTs (a) 5 mg, (b) 10 mg, and (c) 15 mg with continuous addition of glucose in 0.1 M KOH at working potentials of -0.1, 0, 0.1, 0.15, 0.2, and 0.3 V vs. Hg/HgO; Figure S7: CV curves of AuNPs-P-MWCNTs with different HAuCl_4_ concentrations (a) 0.25 mM, (b) 0.5 mM, and (c) 1 mM with 0 mM and 50 mM glucose in 0.1 M KOH at a scan rate of 100 mV·s^−1^ and (d) comparisons of CV curves of AuNPs-P-MWCNTs with different HAuCl_4_ concentrations in 0.1 M KOH containing 50 mM glucose at a scan rate of 100 mV·s^−1^; Figure S8: Chronoamperometric responses of AuNPs-P-MWCNTs with different HAuCl_4_ concentrations (a) 0.25 mM, (b) 0.5 mM, and (c) 1 mM with continuous addition of glucose in 0.1 M KOH at working potentials of −0.1, 0, 0.1, 0.15, and 0.2 V vs. Hg/HgO; Figure S9: CV curves of AuNPs-P-MWCNTs for different discharge times (a) 3 min, (b) 7 min and (c) 9 min with 0 mM and 50 mM glucose in 0.1 M KOH at a scan rate of 100 mV·s^−1^, and (d) comparisons of CV curves of AuNPs-P-MWCNTs with different discharge times in 0.1 M KOH containing 50 mM glucose at a scan rate of 100 mV·s^−1^; Figure S10: Chronoamperometric responses of AuNPs-P-MWCNTs for different discharge times (a) 3 min, (b) 7 min, and (c) 9 min with continuous addition of glucose in 0.1 M KOH at working potentials of −0.1, 0, 0.1, 0.15, and 0.2 V vs. Hg/HgO; Figure S11: CV curves of AuNPs-P-MWCNTs for different discharge voltages (a) 4 kV, (b) 6 kV, and (c) 9 kV with 0 mM and 50 mM glucose in 0.1 M KOH at a scan rate of 100 mV·s^−1^, and (d) comparisons of CV curves of AuNPs-P-MWCNTs with different discharge voltages in 0.1 M KOH containing 50 mM glucose at a scan rate of 100 mV·s^−1^; Figure S12: Chronoamperometric responses of AuNPs-P-MWCNTs for different discharge voltages (a) 4 kV, (b) 6 kV, and (c) 9 kV with continuous addition of glucose in 0.1 M KOH at working potentials of −0.1, 0, 0.1, 0.15, and 0.2 V vs. Hg/HgO; Figure S13: Chronoamperometric responses of AuNPs-C-MWCNTs with continuous addition of glucose in 0.1 M KOH at working potentials of −0.1, 0, 0.1, 0.15, and 0.2 V vs. Hg/HgO.
