# Supplementary material for: Rapid Plasma Synthesis of Gold Nanoparticles Supported on MWCNTs for Electrochemical Detection of Glucose
Source: Materials (Basel). 2025 Jun 28;18(13):3076. doi: 10.3390/ma18133076 (PMC12251022; doi:10.3390/ma18133076)
Supplement: Supplementary file 1 [file materials-18-03076-s001.zip › materials-3658563-supplementary.pdf]

Supplementary Material

Rapid Plasma Synthesis of Gold Nanoparticles Supported on MWCNTs for Electrochemical Detection of Glucose

Qing Yang [1], Yuanwen Pang [1], Hong Li [1,2,*] and Lanbo Di [1,2,*]

[1] College of Physical Science and Technology, Dalian University, Dalian 116622, China
[2] Key Laboratory of Materials Modification by Laser, Ion and Electron Beams, Dalian University of Technology, Ministry of Education, Dalian 116024, China
* Correspondence: lihong10@dlu.edu.cn (H.L.); dilanbo@163.com (L.D.)

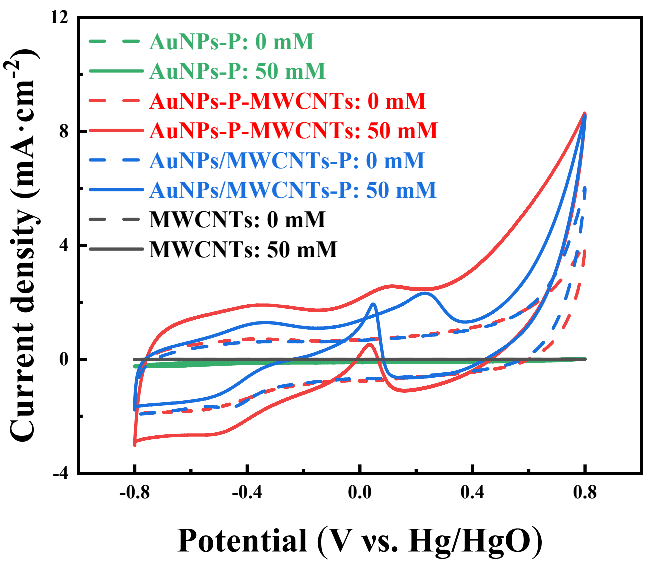

Figure S1. Cyclic voltammetry (CV) curves of AuNPs-P, AuNPs-P-MWCNTs, AuNPs/MWCNTs-P, and MWCNTs with 0 mM and 50 mM glucose in 0.1 M KOH at a scan rate of 100 mV·s^{-1}.

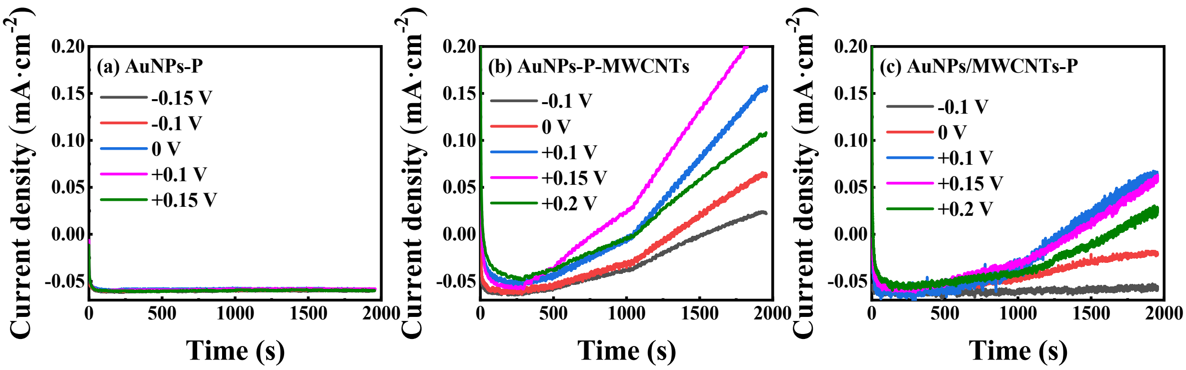

Figure S2. Chronoamperometric responses of (a) AuNPs-P, (b) AuNPs-P-MWCNTs, and (c) AuNPs/MWCNTs-P with continuous addition of glucose in 0.1 M KOH at working potentials of -0.15, -0.1, 0, 0.1, 0.15, and 0.2 V vs. Hg/HgO.

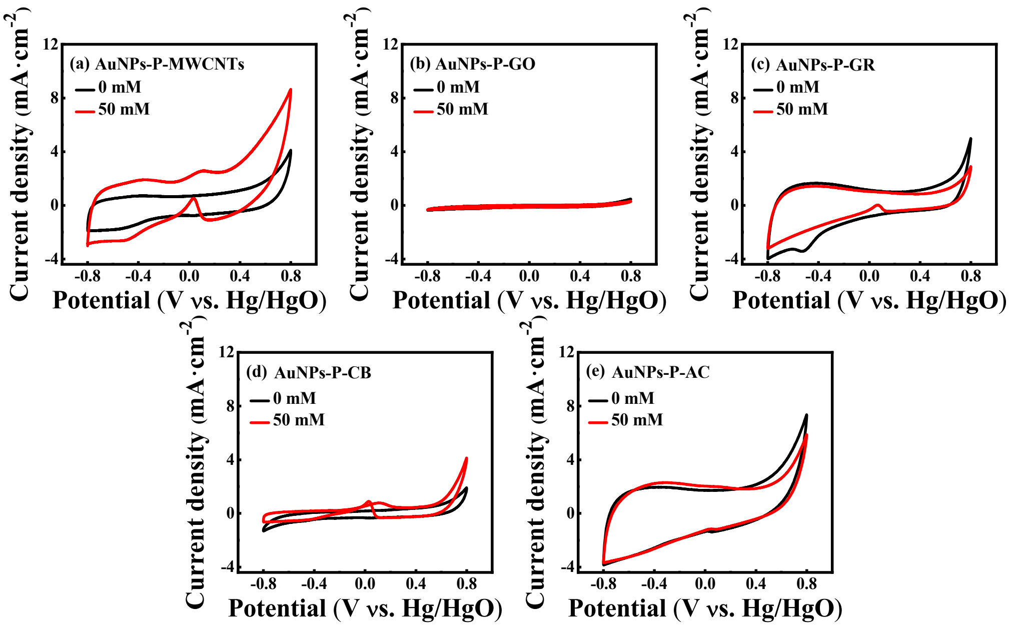

Figure S3. CV curves of (a) AuNPs-P-MWCNTs, (b) AuNPs-P-GO, (c) AuNPs-P-GR, (d) AuNPs-P-CB, and (e) AuNPs-P-AC with 0 mM and 50 mM glucose in 0.1 M KOH at a scan rate of 100 mV·s^{-1}.

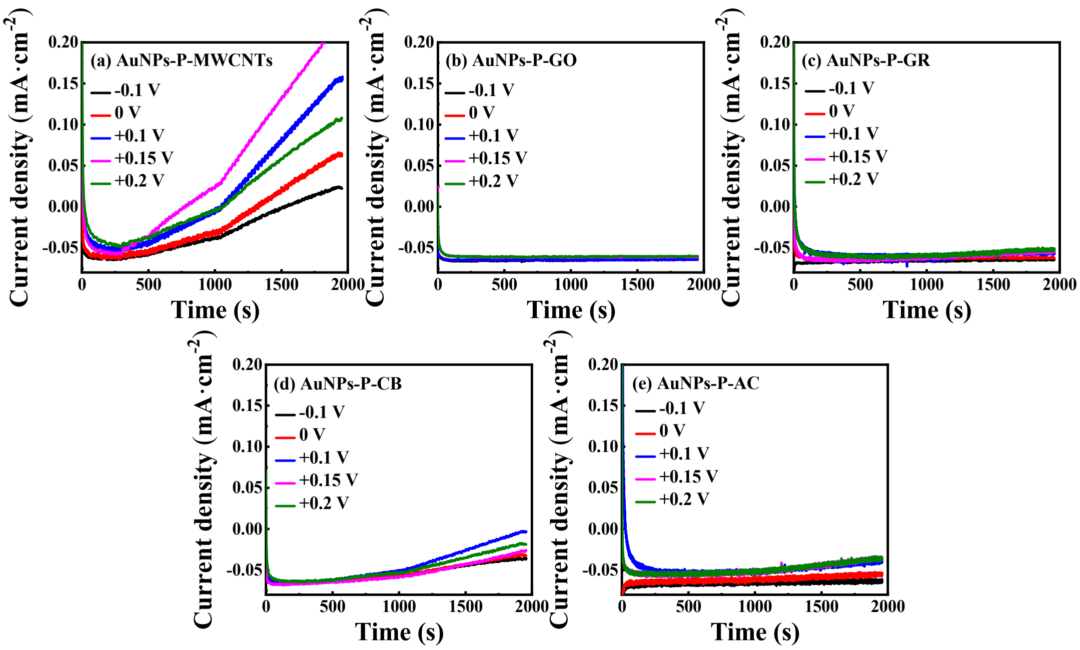

Figure S4. Chronoamperometric responses of (a) AuNPs-P-MWCNTs, (b) AuNPs-P-GO, (c) AuNPs-P-GR, (d) AuNPs-P-CB, and (e) AuNPs-P-AC with continuous addition of glucose in 0.1 M KOH at working potentials of -0.1, 0, 0.1, 0.15, and 0.2 V vs. Hg/HgO.

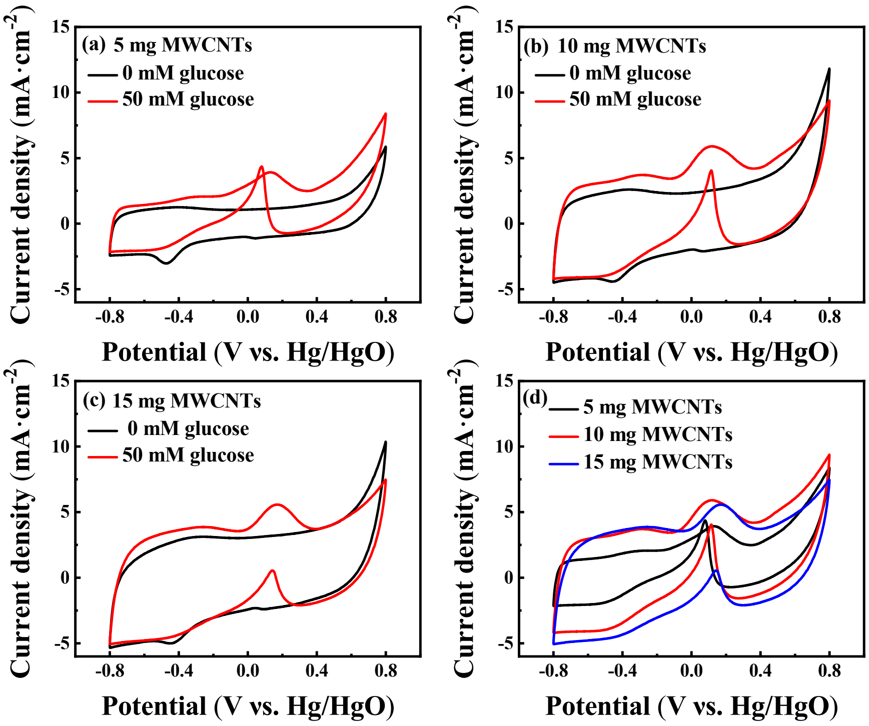

Figure S5. CV curves of AuNPs-P-MWCNTs with different amounts of MWCNTs (a) 5 mg, (b) 10 mg, and (c) 15 mg with 0 mM and 50 mM glucose in 0.1 M KOH at a scan rate of 100 $mV \cdot s^{-1}$, and (d) comparisons of CV curves of AuNPs-P-MWCNTs with different amounts of MWCNTs in 0.1 M KOH containing 50 mM glucose at a scan rate of 100 $mV \cdot s^{-1}$.

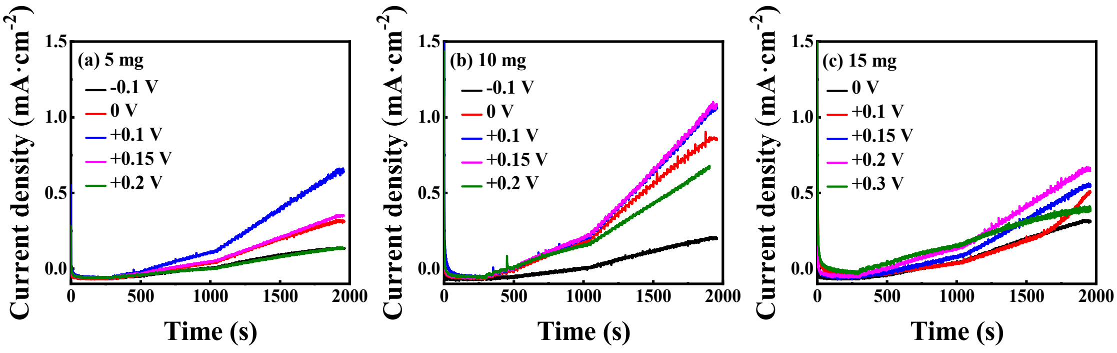

Figure S6. Chronoamperometric responses of AuNPs-P-MWCNTs with different amounts of MWCNTs (a) 5 mg, (b) 10 mg, and (c) 15 mg with continuous addition of glucose in 0.1 M KOH at working potentials of -0.1, 0, 0.1, 0.15, 0.2, and 0.3 V vs. Hg/HgO.

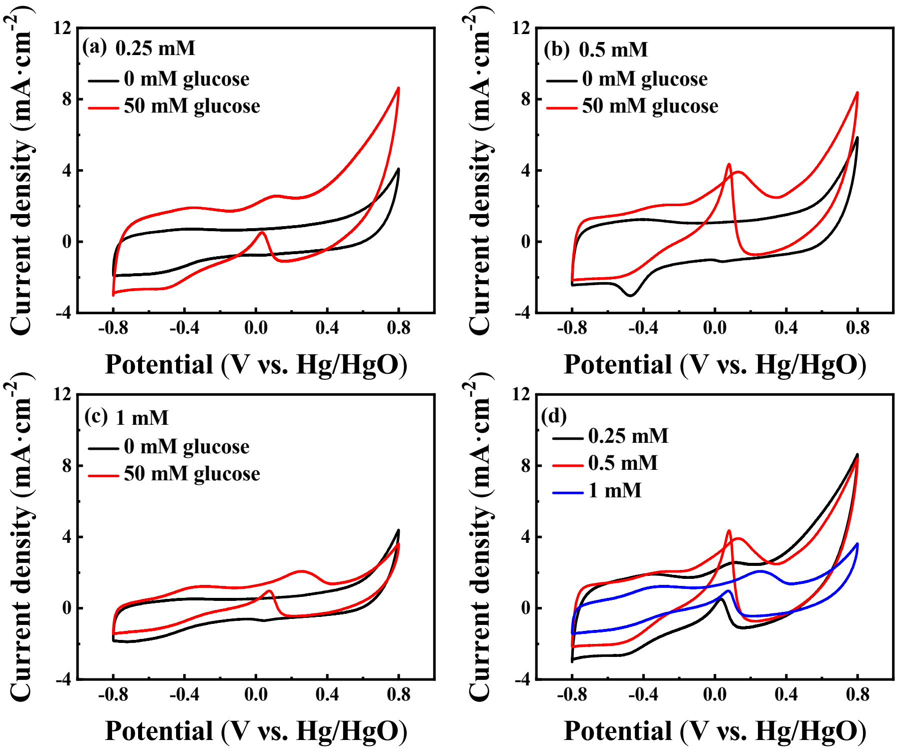

Figure S7. CV curves of AuNPs-P-MWCNTs with different $HAuCl_4$ concentrations (a) 0.25 mM, (b) 0.5 mM, and (c) 1 mM with 0 mM and 50 mM glucose in 0.1 M KOH at a scan rate of 100 $mV \cdot s^{-1}$ and (d) comparisons of CV curves of AuNPs-P-MWCNTs with different $HAuCl_4$ concentrations in 0.1 M KOH containing 50 mM glucose at a scan rate of 100 $mV \cdot s^{-1}$.

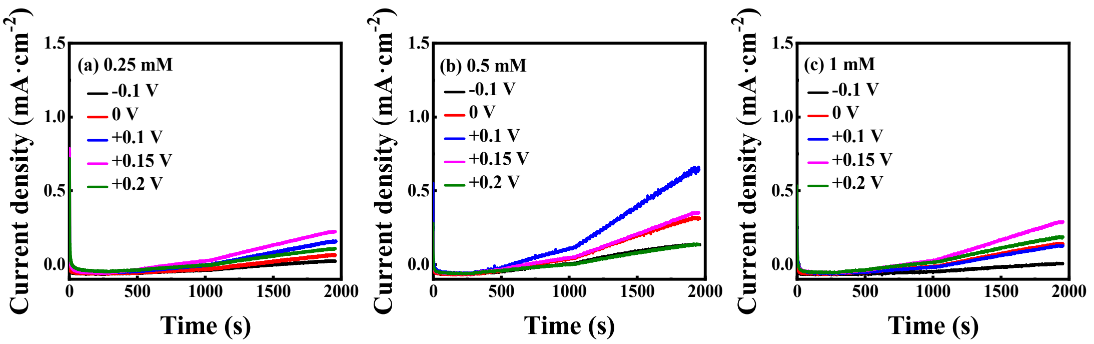

Figure S8. Chronoamperometric responses of AuNPs-P-MWCNTs with different $HAuCl_4$ concentrations (a) 0.25 mM, (b) 0.5 mM, and (c) 1 mM with continuous addition of glucose in 0.1 M KOH at working potentials of -0.1, 0, 0.1, 0.15, and 0.2 V vs. Hg/HgO.

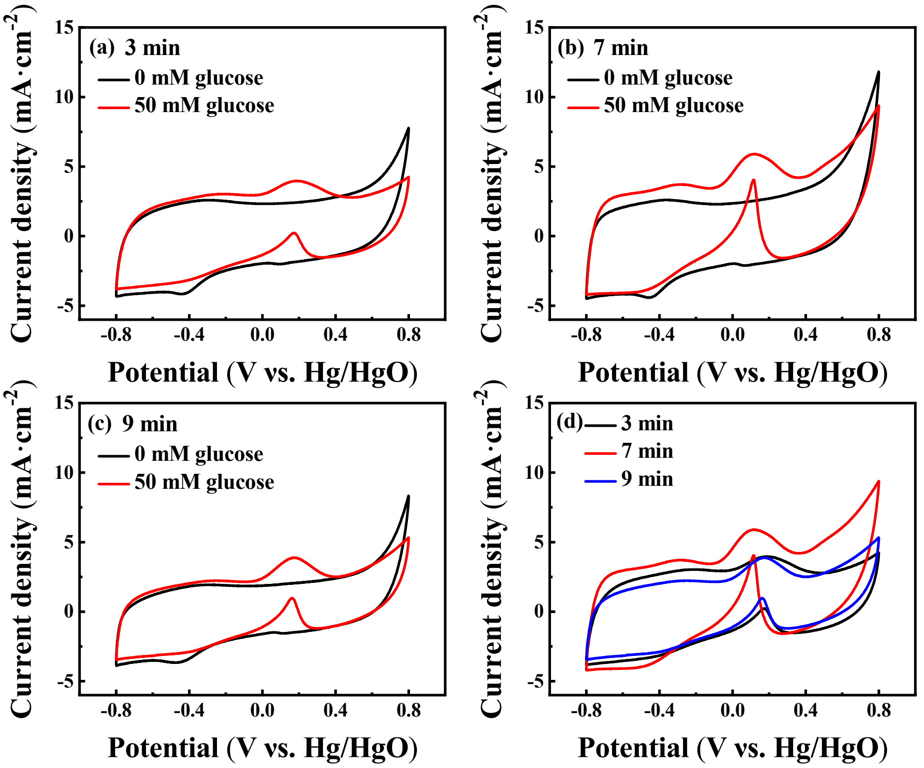

Figure S9. CV curves of AuNPs-P-MWCNTs for different discharge times (a) 3 min, (b) 7 min and (c) 9 min with 0 mM and 50 mM glucose in 0.1 M KOH at a scan rate of 100 $mV \cdot s^{-1}$, and (d) comparisons of CV curves of AuNPs-P-MWCNTs with different discharge times in 0.1 M KOH containing 50 mM glucose at a scan rate of 100 $mV \cdot s^{-1}$.

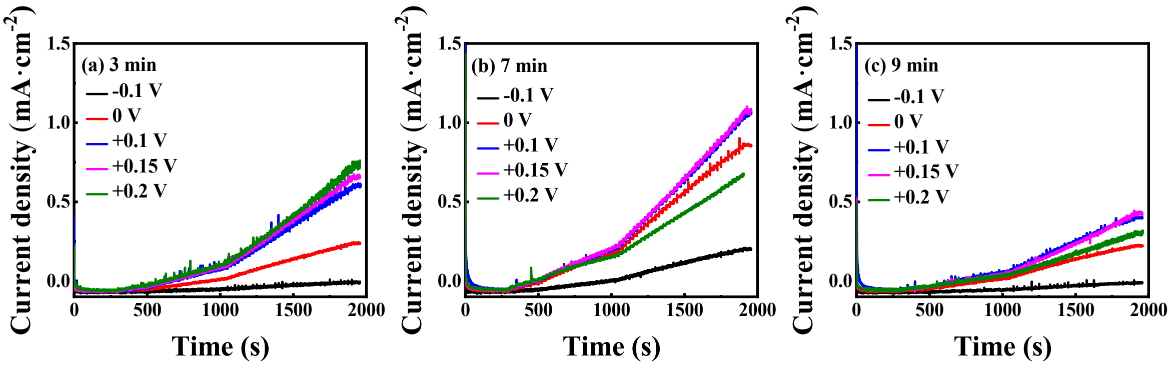

Figure S10. Chronoamperometric responses of AuNPs-P-MWCNTs for different discharge times (a) 3 min, (b) 7 min, and (c) 9 min with continuous addition of glucose in 0.1 M KOH at working potentials of -0.1, 0, 0.1, 0.15, and 0.2 V vs. Hg/HgO.

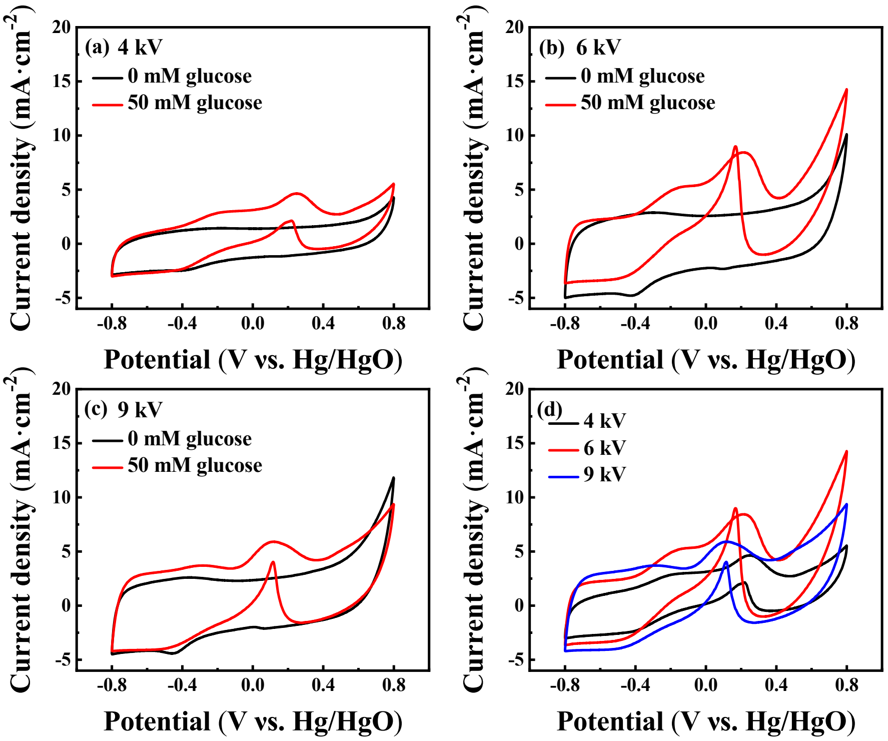

Figure S11. CV curves of AuNPs-P-MWCNTs for different discharge voltages (a) 4 kV, (b) 6 kV, and (c) 9 kV with 0 mM and 50 mM glucose in 0.1 M KOH at a scan rate of 100 $mV \cdot s^{-1}$, and (d) comparisons of CV curves of AuNPs-P-MWCNTs with different discharge voltages in 0.1 M KOH containing 50 mM glucose at a scan rate of 100 $mV \cdot s^{-1}$.

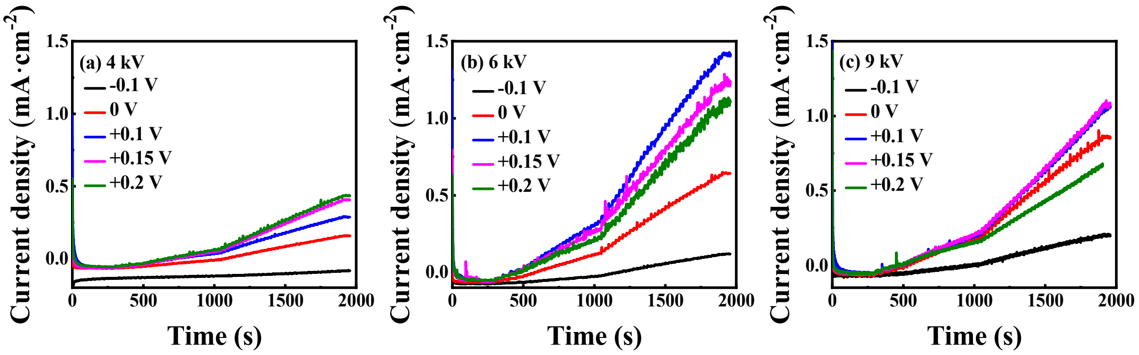

Figure S12. Chronoamperometric responses of AuNPs-P-MWCNTs for different discharge voltages (a) 4 kV, (b) 6 kV, and (c) 9 kV with continuous addition of glucose in 0.1 M KOH at working potentials of -0.1, 0, 0.1, 0.15, and 0.2 V vs. Hg/HgO.

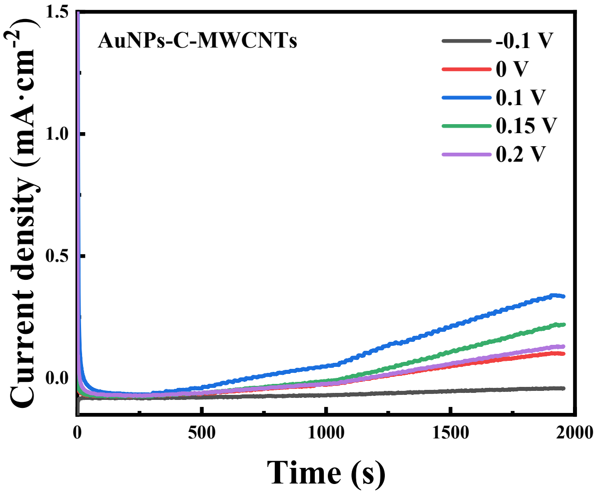

Figure S13. Chronoamperometric responses of AuNPs-C-MWCNTs with continuous addition of glucose in 0.1 M KOH at working potentials of -0.1, 0, 0.1, 0.15, and 0.2 V vs. Hg/HgO.
